# No single host is optimal: effects of fruit species on development and adult performance in *Anastrepha fraterculus* (Diptera: Tephritidae)

**DOI:** 10.3389/finsc.2026.1832786

**Published:** 2026-08-17

**Authors:** Franco Pacelli, María Josefina Ruiz, Francisco Devescovi, Ana Laura Nussenbaum, Micaela Soledad Garbalena, Guillermo Enrique Bachmann, Fabián Horacio Milla, María Teresa Vera, Diego Fernando Segura

**Affiliations:** 1 Instituto de Genética (INTA) – GV IABIMO (CONICET), Centro de Investigaciones en Ciencias Agronómicas y Veterinarias, Instituto Nacional de Tecnología Agropecuaria, Hurlingham, Argentina; 2 Grupo de Investigaciones Biológicas y de Sanidad Agropecuaria, Instituto Superior de Investigaciones Biológicas (Consejo Nacional de Investigaciones Científicas y Técnicas-Universidad Nacional de Tucumán), Tucumán, Argentina; 3 Facultad de Agronomía, Zootecnia y Veterinaria, Universidad Nacional de Tucumán, Tucumán, Argentina; 4 Instituto de Investigación en Ciencias Agrarias y Veterinarias, Universidad del Salvador, Buenos Aires, Argentina

**Keywords:** host preference, host use, larval developmental time, life-history traits, sexual maturation, south American fruit fly, Tephritidae

## Abstract

Many phytophagous insects develop within discrete and non-renewable resources, such as fruits, making host choice a critical determinant of offspring performance. In tephritid fruit flies, host fruit quality can strongly influence development, survival, and adult reproductive traits, yet no single host may optimize all life-history parameters. Here, we evaluated the effects of six host fruit species—native and exotic—on larval development and adult performance of the South American fruit fly *Anastrepha fraterculus*, a highly polyphagous and economically important pest. Under controlled laboratory conditions, we quantified developmental time, pupal survival, adult size, ovary development, male sexual maturation, and starvation resistance. Host species significantly affected all measured traits, revealing pronounced trade-offs among life-history parameters. Guava, a native host, supported the fastest larval development but produced smaller adults with reduced ovary development and lower starvation resistance. Feijoa, another native host, behaved similarly to guava, although pupal mortality was higher and sexual maturation faster than in guava. Peach and plum consistently yielded larger adults with well-developed ovaries and higher overall performance, whereas loquat generally resulted in poorer outcomes across several traits. Grapefruit induced prolonged larval development but promoted early male sexual maturation. Heatmap and PCA analyses confirmed that no single host maximized performance across all traits. These results challenge the assumption that native hosts necessarily confer superior fitness and suggest a decoupling between host use in nature and host suitability under laboratory conditions. Our findings highlight the complexity of host–insect interactions in polyphagous fruit flies and suggest that different host species may be selectively advantageous depending on ecological context and demographic needs. Understanding these trade-offs is essential for predicting population dynamics and improving integrated management strategies for *A. fraterculus*.

## Introduction

Many insect species lay eggs in discrete, isolated resource patches—such as fruits, seeds, or flower heads—that serve as the sole developmental environment for their offspring. In these species, larvae cannot move to a different patch once hatched, so maternal site selection critically determines progeny fate (1). The quality of these patches directly affects key early-life traits, such as larval survival, developmental time, and pupal size or weight. These, in turn, shape later-life traits such as adult survival, lifespan, female fecundity, and male mating success (2). The role of females in choosing suitable hosts for their progeny has been studied as a way of testing the preference–performance (or “mother knows best”) hypothesis, which postulates that females preferentially oviposit on hosts that maximize offspring fitness (3–6).

True fruit flies (Diptera: Tephritidae) are among the most economically significant insect pests worldwide (7, 8). Their impact stems from the damage caused by larval feeding inside ripening fruits, which renders them unmarketable. Female fruit flies possess a specialized ovipositor, sometimes serrated, that allows them to pierce fruit skin and deposit eggs within. After hatching, larvae are confined to the fruit, which, as mentioned before, makes female fruit choice critical to offspring survival and fitness (9). Due to their broad host range, including many commercial crops, fruit flies are not only major agricultural pests but also serve as model organisms for studying insect-plant interactions. In particular, studies on host preference and host suitability, addressing the mother-knows-best hypothesis, can illustrate how female choice shapes progeny performance and, ultimately, pest dynamics. In Tephritidae, such studies have involved a limited number of species, like *Anastrepha ludens* (Loew), *Bactrocera dorsalis* (Hendel), and *Ceratitis capitata* (Wiedemann). In *B. dorsalis*, oviposition preference closely matches offspring performance, with ripe and fully-ripe mangoes being preferred and supporting higher larval survival and faster development than unripe fruits (10). On the other hand, in *A. ludens*, this relationship is weak and inconsistent across host species (11), and in *C. capitata* preference generally matched larval performance for fruit ripeness, but not consistently for all the fruit species evaluated (12, 13).

Host suitability in Tephritidae fruit flies has been studied through both laboratory experiments and field surveys, showing that host plant species strongly influence oviposition preference and key fitness traits such as larval survival, development, and adult performance. The reasons that explain the differential performance among host species of certain Tephritidae species remain largely unknown. In other groups of phytophagous insects, there is a general agreement that native host plants allow for better development compared with introduced plant species (14, 15). However, in certain cases, fitness improved on novel hosts, particularly in polyphagous and invasive insects or when colonizing close relatives of ancestral host plants (14). Besides their common evolutionary past, critical aspects that determine the quality of a host plant include its nutritional quality (16, 17). As an example, in *D. melanogaster*, protein-poor and carbohydrate-rich diets have been shown to extend lifespan (18). Despite these insights from other phytophagous and polyphagous insects, including dipterans, it remains unclear in Tephritidae which factors primarily determine host suitability, and whether evolutionary history or fruit nutritional traits play the dominant role in shaping fly performance.

Despite the vast number of studies that assessed the performance of fruit fly species in different host plants, a comparison between native and exotic fruit species is missing. Studies that recorded fruit infestation under natural conditions suggested that fruit fly species are more commonly associated with native host species than with exotic species, which suggests they may be better adapted to native hosts (19–23). However, certain fruit species serve as suitable hosts for multiple tephritid fruit fly species, regardless of their evolutionary past. For example, mango (*Mangifera indica*) is commonly infested by species that are native to different continents. Similarly, guava (*Psidium guajava*) is frequently targeted by multiple fruit fly species that evolved in areas where guava is not a native species. This suggests that the relationship between fruit origin and host suitability is not obvious and may be influenced by factors such as fruit quality, insect sensorial biases, and ecological interactions. Therefore, while general trends can be observed, host suitability is species-specific and context-dependent.

*Anastrepha fraterculus* (Wiedemann), commonly known as the South American fruit fly, is a highly polyphagous insect pest native to South America (24). This species is considered a cryptic species complex (25, 26), which exhibit moderate to high sexual isolation (27–32). In Argentina, only a single morphotype, called *A. fraterculus* sp. 1 or Brazilian morphotype 1, has been reported (25). Although it has been reported that *A. fraterculus* infests a broad range of over 177 fruit species, including economically significant crops (19, 20) a deep literature review shows that host use is also different among morphotypes (22). Its host range and variation pose substantial challenges to fruit production and international trade due to stringent quarantine regulations (24). In addition, the high fecundity and multiple generations per year that characterize *A. fraterculus*, exacerbate its impact on agriculture.

Understanding host preference and performance in *A. fraterculus* is essential for uncovering how populations establish and expand. Given the challenges in assessing oviposition preference, especially considering the extensive host range, offspring performance may also serve to generate specific predictions under the preference-performance hypothesis. Previous work suggest that *A. fraterculus* predominantly infests native fruits (33). However, de Souza et al. (34) found no such association when measuring the number of eggs laid on fruits from different species. These contrasting results highlight the complexity of host selection in this species, likely due to the wide range of host plants and the genetic and behavioral variability across *A. fraterculus*´s broad geographic distribution (35–37). In this study, we evaluated the performance of *A. fraterculus* sp. 1 offspring on a selected group of host species that included native as well as exotic species from different taxonomic groups, under laboratory conditions. We hypothesized that offspring performance is strongly influenced by host species. We predicted that both larval and adult traits would vary depending on the rearing fruit, and accordingly, we examined life-history parameters across six different host species.

## Materials and methods

### Insects

Females of the Horco Molle laboratory colony (HM) were used in all tests. HM colony was established with wild pupae recovered from wild-infested guava collected in Horco Molle, Tucumán, Argentina (26° 49’ 0” S/65° 19’ 0” W). Pupae were maintained under laboratory conditions (25 °C ± 1 °C and 70% ± 10% RH) until emergence, and adults were then reared under artificial conditions. Adults were kept in translucent, plastic cages (40 × 20 × 20 cm) and were provided with water and food [sugar, hydrolyzed brewer’s yeast (Yeast Hydrolysate Enzymatic; MP Biomedicals, Aurora, OH, USA), and hydrolyzed corn (Gluten Meal; Arcor, Tucumán, Argentina) in 4:1:1 ratio] (38). For the first two generations, mango was used as oviposition substrate. After F2, flies were offered artificial oviposition devices made of plastic vials (10 ml) filled with tap water colored with a red food dye (Fleibor; Tablada, Buenos Aires, Argentina) and covered with a thin layer of Parafilm-M (Pechiney Plastic Packaging, Chicago, IL, USA). Females laid eggs piercing through the parafilm layer. The vials were placed inside the rearing cage and left there for 24 h. After this time, the vials were removed, and the eggs were collected by filtering the colored water. Collected eggs were transferred to an artificial larval diet. Larval rearing followed standard procedures using an artificial diet based on non-hydrolyzed brewer’s yeast (CALSA^®^, El Manantial, Tucumán, Argentina), wheat germ, sugar, and agar (39). Females from this colony were used in the experiments from generation 17 to generation 34, and they were 15 ± 1 days old. Because this species reaches sexual maturity around 10 days after emergence (40, 41), females were assumed to be mated when used in the experiments.

### Host fruit species

The effect of the host species on the development of *A. fraterculus* was studied by comparing six fruit species. These species have all been reported to be attacked by *A. fraterculus* in nature (42–44). Host species were selected based on both ecological and practical criteria. Our selection aimed to include a range of native (2) and introduced (4) host species, as well as representatives from different taxonomic genera (5) and families (3). We also chose host species that can be collected or bought from the market in large numbers all year round, so as to carry out an adequate number of replicates. Three host fruit species were obtained in local markets: grapefruit (*Citrus x paradisi* Macfad, Rutaceae, exotic), peach (*Prunus persica* L., Rosaceae, exotic), and plum (*Prunus domestica* L., Rosaceae, exotic). The other host fruit species were collected from wild trees. These included loquat (*Eriobotrya japonica* (Thunb.) Lindl., Rosaceae, exotic) (34°36’59.5”S 58°39’39.7”W), guava (*Psidium guajava* L. Myrtaceae, native) (34°38’38.5”S 58°38’55.1” W), and feijoa [*Feijoa sellowiana* (O. Berg) Burret., Myrtaceae, native] (34°35’12.2”S 58°28’16.0” W). For each host fruit species, the same varieties were used in all trials. In the case of fruit obtained from the wild trees, fruits without signs of natural infestation (decolored and soft patches or punctures on the fruit surface) were collected. In each sampling, a group of fruits was incubated for 3 weeks to determine if there were any infested fruit (control fruit). Replicates where natural infestation was detected were discarded from the experiment. In all cases, only ripe, undamaged fruits were used, selected based on their color and firmness.

### Fruit infestation

Fruits were exposed to 200 sexually mature flies (100 females and 100 males) for 3 h in 30 L infestation cages under no-choice conditions. These conditions aimed at keeping low infestation densities, so intraspecific competition did not affect the quality of the hosts, and were chosen based on previous works done by our group (40, 45). All experiments were conducted under controlled environmental conditions (25 °C ± 1 °C, 60% ± 10% RH, and 12L:12D photoperiod). Due to differences in fruit size among host species, the volume of fruit offered to females was standardized across cages (which was approximately 1,200–1500 cm^3^ of fruit per cage). Each fruit was measured at its equatorial diameter, and this value was used to estimate the radius of a perfect sphere. Fruit volume was then calculated as V = (4/3)×π×r^3^; this parameter was used to equalize the volume of fruit offered to the females at the time of infestation. For instance, the volume of one grapefruit is approximately equivalent to the volume of five loquats or three plums. After exposure, infested fruits were individually transferred to plastic containers (125 mL) perforated at the bottom to facilitate the passage of the larvae to the pupation substrate. Each container was then placed inside a larger plastic container (250 mL) containing a layer of vermiculite as a pupation substrate. Containers were maintained in incubators under the same temperature and relative humidity conditions described above. After 10 days, the vermiculite was sieved to recover the pupae; a procedure that was repeated every 24 h until no pupae were recovered. Pupae from multiple fruits and infestation events within each host species were grouped, and emerging adults were randomly selected for further measurements, maybe introducing a certain (yet tolerable) lack of independence among replicates.

### Recorded parameters

#### Developmental time (egg–pupa)

Developmental time was defined as the number of days from infestation to pupation and was recorded for each host species. The number of pupae collected each day was used to calculate egg-to-pupa developmental time as a weighted average (see Data Analysis). The number of experimental units (one container with infested fruit) varied among host species: guava (n = 51), feijoa (n = 51), loquat (n = 41), grapefruit (n = 36), peach (n = 34), and plum (n = 99).

#### Adult emergence

Pupae collected from each container were transferred daily to 3-L glass containers and maintained there until adult emergence. Containers were maintained under previously described controlled environmental conditions and monitored daily. Upon emergence, adult flies were assigned to the different experiments described below. The number of emerged adults was recorded, and the proportion of adult emergence (pupal survival) was calculated for each host fruit species (number of emerged adults/total number of pupae) in each container. The number of experimental units (one container with pupae) varied among host species: guava (n = 26), feijoa (n = 51), loquat (n = 41), grapefruit (n = 21), peach (n = 17), and plum (n = 45).

#### Wing length

**Figure 1:**
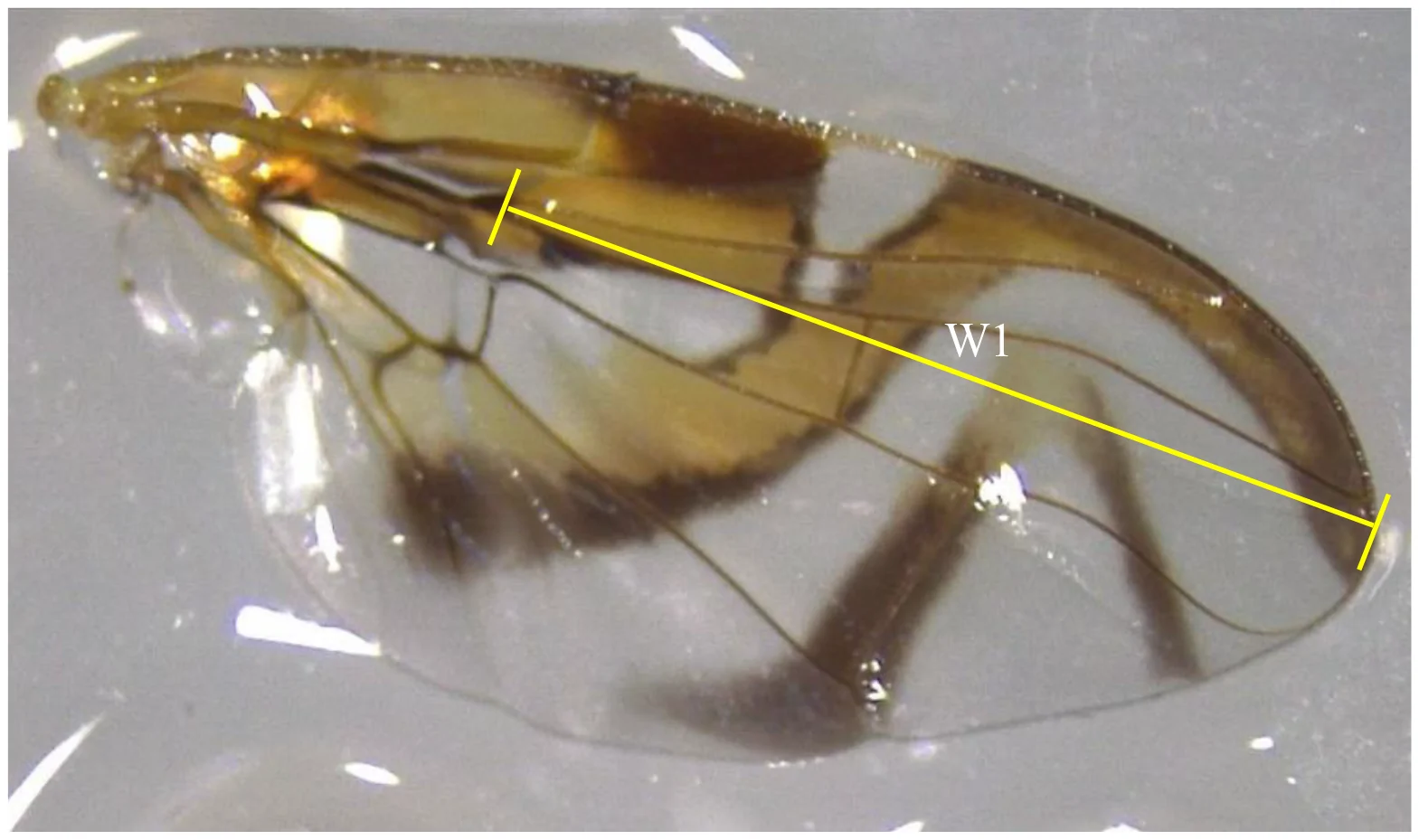
Dorsal view of the right wing of *Anastrepha fraterculus* showing the segment used to represent wing length (W1).

To evaluate whether host species affected fly size, wing length (mm) was used as an estimator of adult size, with one wing measured per female. Wing length was considered a proxy for structural body size, as it has been commonly used in tephritid species (46–51). The right wing was removed and placed fully extended on a microscope slide. The wings were photographed under stereomicroscope (4x, Leica, Wetzlar, Alemania; software Leica Acquire LAZ-EZ-3.4). The length of the right wing of each fly was measured from the basal bifurcation of R2 + 3 and R4 + 5 to the end of the apex of vein R4 + 5 (**Figure 1**) using the MShot Image Analysis System software (v 1.0, Micro-shot Technology Co., Guangzhou, China). The number of wings varied among host species: guava (n = 49), feijoa (n = 96), loquat (n = 48), grapefruit (n = 26), peach (n = 58), and plum (n = 98).

#### Ovary length

**Figure 2:**
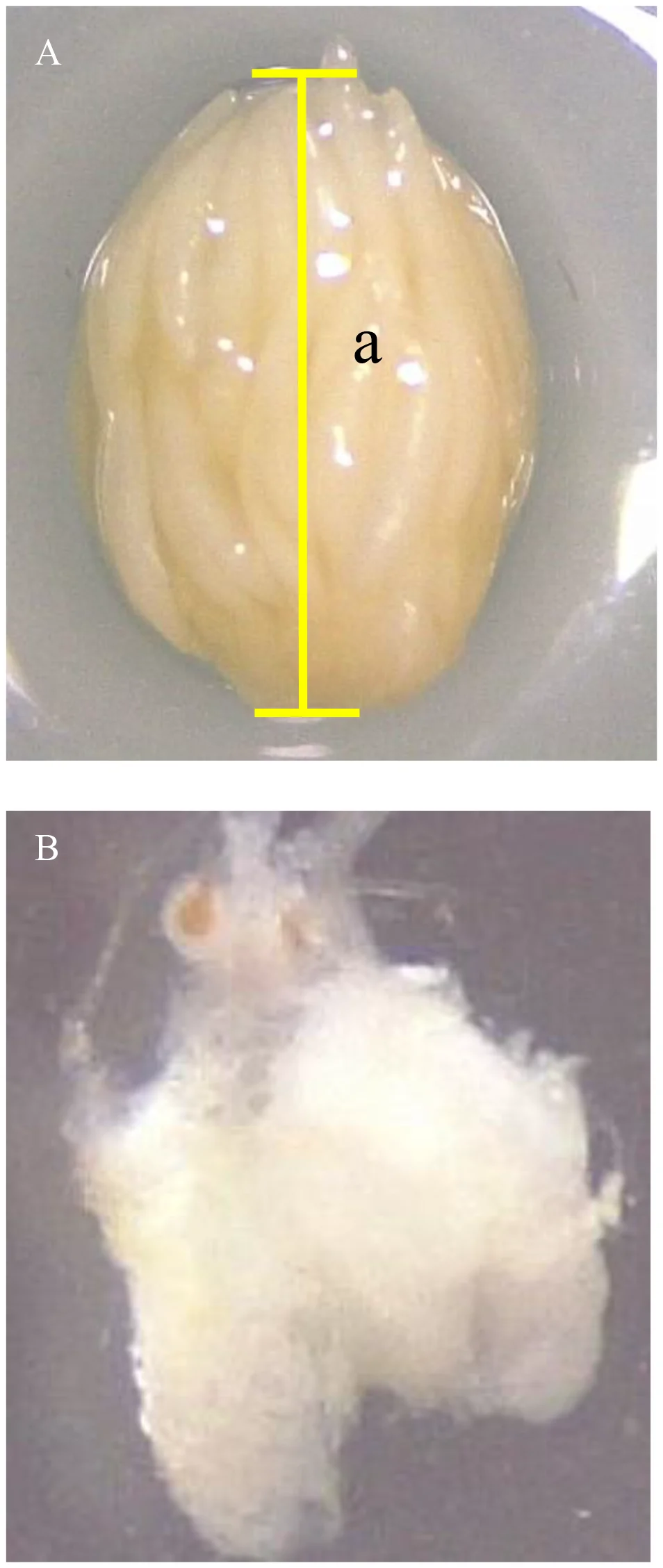
Ovary condition in 10 days-old *Anastrepha fraterculus* females. **(A)** Normal ovary indicating length (a). **(B)** Atrophied ovary.

Ovary length was measured following the methodology described by Hou et al. (52). Females emerging from different host fruit species were transferred to 3-L glass containers and provided with water and an adult diet (sugar and brewer’s yeast in a 3:1 ratio) *ad libitum*. After 10 days, the time at which *A. fraterculus* females reach sexual maturity (53), individuals were preserved in 70% ethanol at −20 °C until dissection. During dissection, the ovaries of each female were removed, placed on a slide, and visualized under a stereomicroscope (4x) equipped with image capture software (Leica Acquire LAZ-EZ-3.4). Photographs were used to measure the maximum ovary length of each female and were taken on one ovary (selected at random) per female (**Figure 2**). This measurement was used to assess the effect of host fruit on ovary length, an indicator of female fecundity potential (52). The number of ovaries measured per host species was: guava (n = 30), feijoa (n = 78), loquat (n = 17), grapefruit (n = 28), peach (n = 39), and plum (n = 90).

In addition, for each host species, the number of developed and atrophied ovaries (i.e., ovaries that did not develop) was recorded, and the proportion of atrophied ovaries was calculated. The number of experimental units (ovaries recorded) varied among host species: guava (n = 94), feijoa (n = 188), loquat (n = 83), grapefruit (n = 53), peach (n =113), and plum (n = 186).

#### Sexual maturation of males

To evaluate whether the host species affects the sexual maturation of males, the age at which males start to release sexual pheromones was recorded for males reared in each host species (41). When *A. fraterculus* males are sexually mature, they perform a series of species-specific, stereotyped courtship behaviors, which typically involve pheromone release by exposing the salivary glands and anal pouch coupled with bursts of rapid wing-fanning to enhance pheromone dispersion (54–56). After emergence, groups of five males were used to mimic leks normally observed in nature to attract females (57, 58). The five males were placed in 3-L glass containers with water and food (sugar and hydrolyzed brewer´s yeast, 3:1 ratio). Twenty-four hours before the experiment, males were marked with a dot of water-based paint on the notothorax. Different colors were used to distinguish those males performing the calling behavior. The containers with the groups of males were transferred to incubators under controlled environmental conditions and light cycle (25 °C ± 1°C, 70% ± 10% RH, 12L:12D). The experiment started when males were one day old. The day each male exhibited its first sexual courtship behavior was recorded. The observations were made daily, from 8:30 (after dawn) to 11:00 am, a period during which most copulations occur in this species (57). The number of experimental units (analyzed males) varied among host species: guava (n = 44), feijoa (n = 61), loquat (n = 20), grapefruit (n = 35), peach (n =30), and plum (n = 49). The experiment ended when all males had initiated calling.

#### Starvation resistance

To evaluate the effect of host species on the starvation resistance of adult flies, newly emerged males and females were individually placed in 15 mL Falcon tubes sealed with a moist cotton to ensure water availability. The cotton was re-moistened every 8 hours. Tubes were maintained in incubators under controlled environmental conditions (see above). Flies were monitored twice a day (08:00 and 16:00). Upon detection of mortality, sex, treatment, and time (days since emergence) were recorded. The assay continued until all individuals had died. Mortality was confirmed when a fly exhibited no response to mechanical stimulation with a fine brush. The number of males and females varied among host species: guava (females: n = 59; males: n = 48), feijoa (females: n = 96; males: n = 120), loquat (females: n = 55; males: n = 53), grapefruit (females: n = 40; males: n = 49), peach (females: n = 55; males: n = 54), and plum (females: n = 166; males: n = 148).

### Data analysis

The effect of host species on the egg-pupa developmental time was analyzed using a weighted average for each fruit as to include the contribution of each pupa to the developmental time. A linear model was used to analyze this response variable. The host species was considered a fixed factor. The residuals were examined for normality and homoscedasticity. Since the assumptions were not met, a generalized linear model (GLM) was applied with a gamma distribution and a log link function. Comparison of means was performed with Tukey’s test (*emmeans* package).

The proportion of adult emergence was analyzed using a GLM with a binomial error distribution and a logit link function. Because overdispersion was detected, a quasibinomial distribution was used. Host species was included as a fixed factor and the proportion of emerged flies as the response variable. *Post hoc* comparisons among means were performed using Tukey’s test (*emmeans* package).

To assess the effect of host species on wing length we used a linear model with host species as the fixed factor and wing length as the response variable. Residuals were tested for normality and homoscedasticity. Since the assumptions were not met, a GLM with a gamma distribution and a log link function was applied. Pairwise comparisons were made using Tukey’s test (*emmeans* package).

To evaluate the effect of host species on ovary length, a linear model was fitted. Host species was included as the fixed factor and ovary length as the response variable. Residuals were tested for normality and homoscedasticity. Since the assumptions were not met, a GLM with a gamma distribution and a log link function was applied. Pairwise comparisons were performed using Tukey’s test (*emmeans* package).

The effect of host species on the proportion of atrophied ovaries was analyzed using a GLM with a binomial error distribution and a logit link function. Host species was included as a fixed factor and the proportion of atrophied ovaries as the response variable. Due to overdispersion, a quasibinomial distribution was applied. Pairwise comparisons were performed using Tukey’s test (*emmeans* package).

The effect of host fruit species on the onset of calling behavior (measured in days), was analyzed using a parametric survival model with a Weibull distribution (survival package). Host fruit species was included as a fixed factor, and the time to calling was the response variable. Model significance was assessed using likelihood-ratio chi-square tests. Kaplan–Meier curves were used to visualize the cumulative probability of calling over time for each host fruit species (*survminer* package). Pairwise comparisons among host species were conducted using Cox proportional hazards models (coxph), followed by Tukey-adjusted *post hoc* tests implemented with the multcomp and multcompView packages.

Survival under starvation was analyzed using a parametric survival model with a Weibull distribution (survival package) to assess the effect of host fruit species, sex, and their interaction on nutritional stress tolerance. Host fruit species, sex, and their interaction were included as fixed factors, and survival time (measured in days) was the response variable. The model significance was assessed using likelihood-ratio chi-square tests. Kaplan–Meier survival curves were used to visualize survival patterns over time among host fruit species (*survminer* package). Pairwise comparisons among host fruit species were conducted using Cox proportional hazards models (coxph), followed by Tukey-adjusted *post hoc* tests implemented with the *multcomp* and *multcompView* packages.

In addition, a heatmap analysis was performed to integrate the biological traits analyzed above. Model-adjusted means, estimated proportions, or median values were combined into a single matrix and standardized by row (z-scores) to allow comparison of relative performance across host fruits. For traits where lower values indicate better biological performance (developmental time, proportion of atrophied ovaries, and onset of calling behavior), standardized values were inverted prior to visualization. Heatmaps were generated using the pheatmap package in R.

To explore the relationship between host fruit nutritional composition and offspring life-history traits, a principal component analysis (PCA) was performed using the mean values of all nutritional and biological variables for each host species. Nutritional data were obtained from previously published information on the same host fruits, whereas biological traits were derived from the present study. Variables were standardized before analysis. PCA results were visualized using the *factoextra* package.

In all tests, the statistical significance level was set at α < 0.05. All data analyses were performed using the R v. 4.5.2 statistical environment (59).

### Nutritional composition of the host fruit species

Nutrient content data for six host fruit species were compiled from the U.S. Department of Agriculture (USDA) Agricultural Research Service: National Nutrient Database for Standard Reference (2015). Data included water, protein, total lipids, ash (total mineral content of the fruit), total carbohydrates (by difference), total sugars, and dietary fiber. The complete data set is provided in **Table 1**.

**Table 1:** Nutrient contents obtained from the USDA, National Nutrient Database for Standard Reference for six host fruit species (g per 100 g of pulp).

Nutrients	Host fruit species					
	Guava	Feijoa	Loquat	Grapefruit	Peach	Plum
Water	80.8	83.3	86.7	88.6	88.3	87.23
Protein	2.55	0.71	0.43	0.77	0.91	0.7
Total lipid (fat)	0.95	0.42	0.2	0.14	0.27	0.28
Ash	1.39	0.38	0.5	0.22	0.43	0.74
Carbohydrate, by difference	14.3	15.2	12.1	10.66	10.1	11.42
Sugars, total	8.92	8.2	10.4	6.89	8.39	7.96
Fiber, total dietary	5.4	6.4	1.7	1.6	1.5	1.4

## Results

### Developmental time

**Figure 3:**
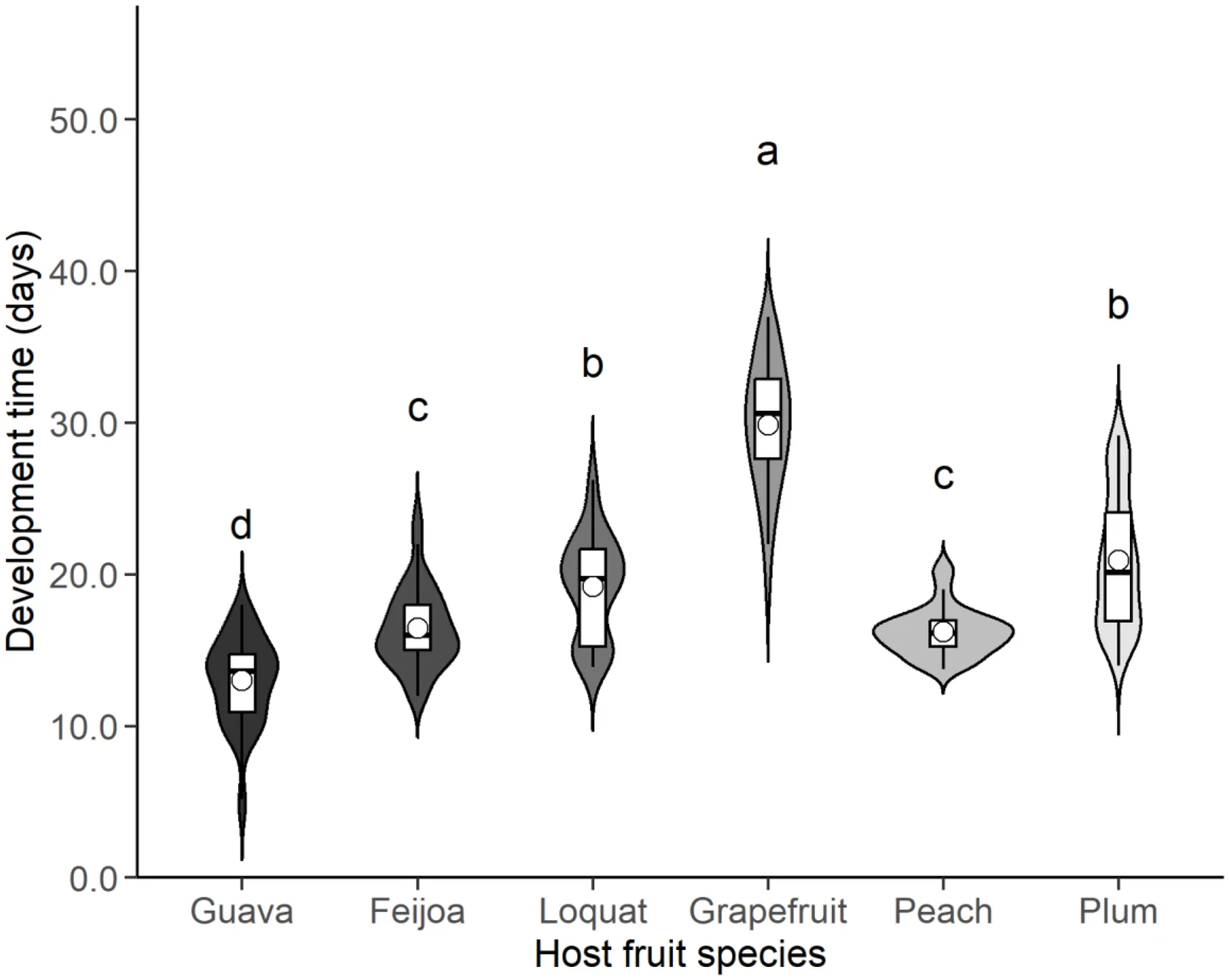
Developmental times (egg–pupa, in days) of *Anastrepha fraterculus* reared in different host species. The experiment involved six host species: *Psidium guajava* (guava), *Acca sellowiana* (feijoa), *Eriobotrya japonica* (loquat), *Citrus x paradisi* (grapefruit),, *Prunus persica* (peach), and *Prunus domestica* (plum). The data are presented as means ± SE. Different letters indicate statistically significant differences among means (p < 0.05).

The developmental time (days) of *A. fraterculus* from oviposition to pupation differed among host fruit species (χ² = 17.89; d.f. = 5, 306; p < 0.001). Developmental time was significantly longer for larvae fed on grapefruit (29.9 ± 0.3 days), followed by plum and loquat (20.9 ± 0.38 and 19.2 ± 0.55 days, respectively), and then peach and feijoa (16.2 ± 0.51 and 16.5 ± 0.42 days, respectively) (**Figure 3**). Developmental time was shorter for larvae fed on guava (13.0 ± 0.33 days) (**Figure 3**).

#### Adult emergence

**Figure 4:**
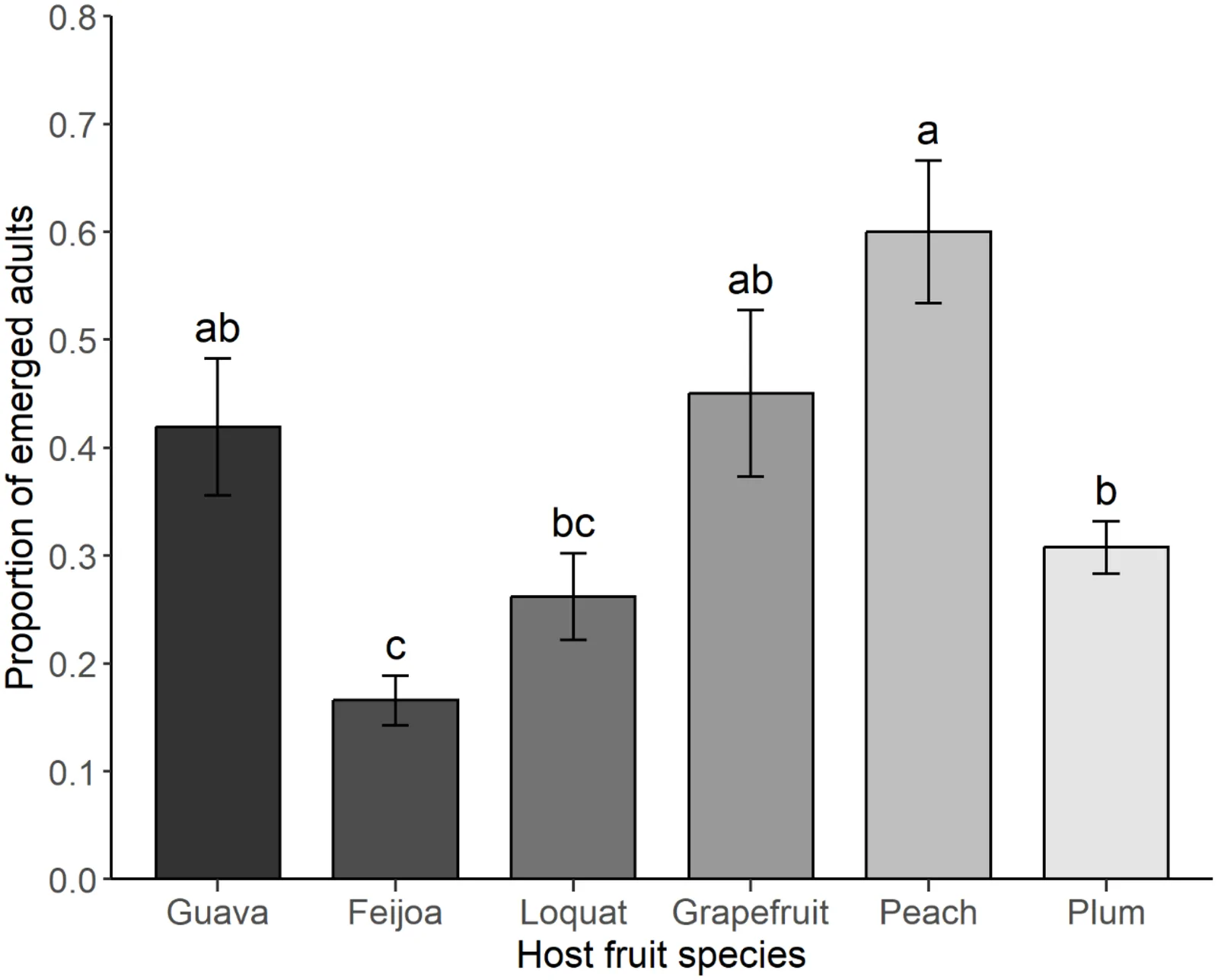
Proportion of emergence of *Anastrepha fraterculus* adults reared on different host fruit species including *Psidium guajava* (guava), *Acca sellowiana* (feijoa), *Eriobotrya japonica* (loquat), *Citrus x paradisi* (grapefruit),, *Prunus persica* (peach), and *Prunus domestica* (plum). Proportion (± S.E.) are shown for each host species. Different letters indicate statistically significant differences among means (p < 0.05).

The proportion of emerged *A. fraterculus* adults was significantly affected by host species (F = 10.93; d.f. = 5, 195; p < 0.001). Emergence was highest in flies reared on peach (0.60 ± 0.066), followed by those reared on grapefruit and guava (0.45 ± 0.077 and 0.42 ± 0.063, respectively). Intermediate values were observed in flies reared on plum and loquat (0.31 ± 0.024 and 0.26 ± 0.040, respectively), whereas the lowest emergence was recorded in flies reared on feijoa (0.17 ± 0.023) (**Figure 4**).

### Wing length

**Figure 5:**
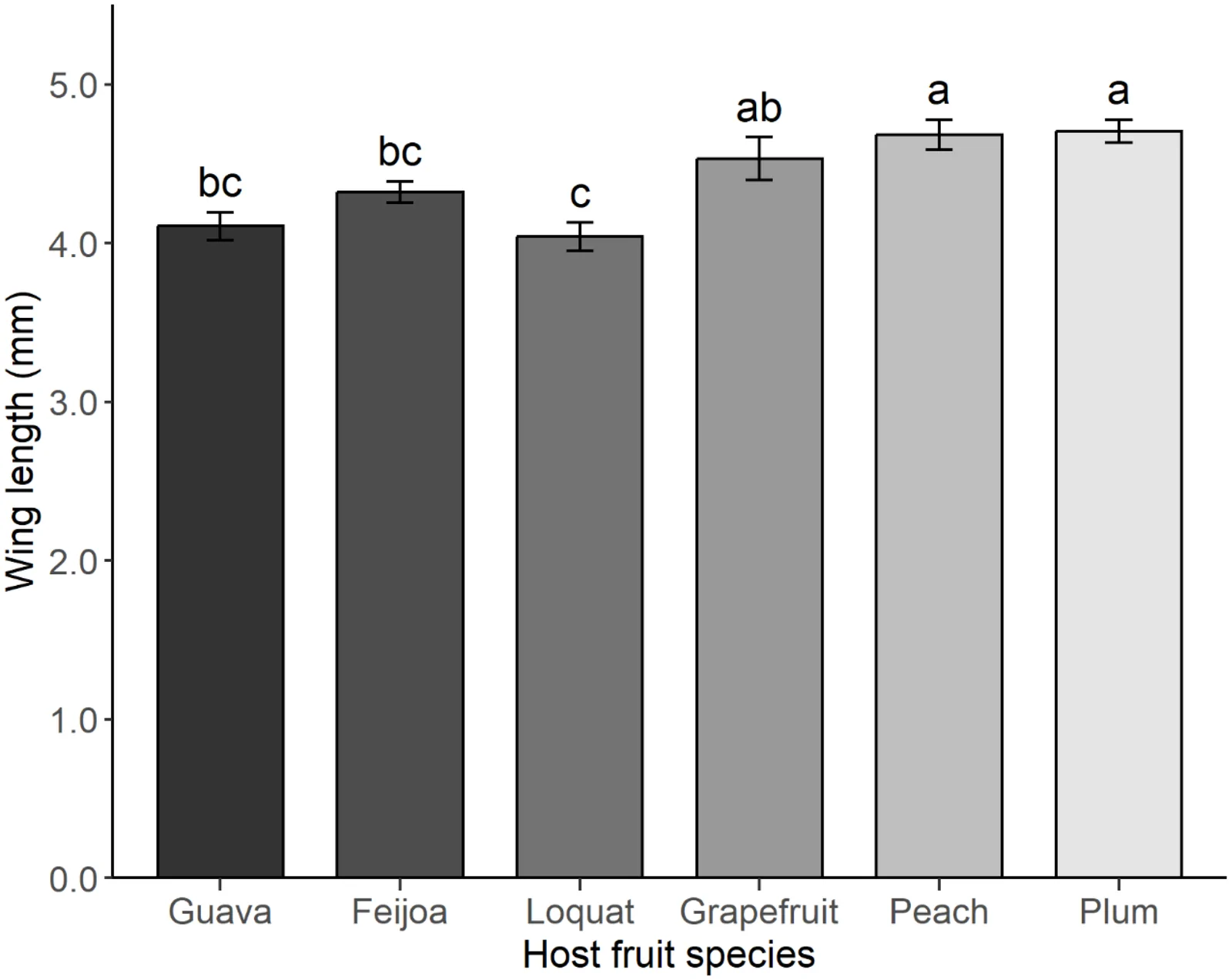
Wing length of *Anastrepha fraterculus* females that developed in different host species including *Psidium guajava* (guava), *Acca sellowiana* (feijoa), *Eriobotrya japonica* (loquat), *Citrus x paradisi* (grapefruit),, *Prunus persica* (peach), and *Prunus domestica* (plum). Mean (± S.E.) is shown in mm for each host species. Different letters indicate statistically significant differences among means (p < 0.05).

Wing length of *A. fraterculus* was affected by the host species where the larva developed (likelihood ratio χ²= 1.286; d.f. = 5; p < 0.001). Female wing length was significantly greater in individuals reared on plum (4.71 ± 0.07 mm) and peach (4.68 ± 0.09 mm) than in those reared on feijoa, guava, and loquat. Females reared on grapefruit exhibited intermediate wing lengths (4.53 ± 0.13 mm) and did not differ significantly from those reared on plum and peach, nor from those reared in feijoa and guava. Wing length was lowest in females reared on loquat (4.04 ± 0.09 mm), being significantly smaller than in females reared on plum, peach, and grapefruit, and not differing significantly from those reared in feijoa (4.32 ± 0.07 mm) and guava (4.11 ± 0.09 mm) (**Figure 5**).

### Ovary development

**Figure 6:**
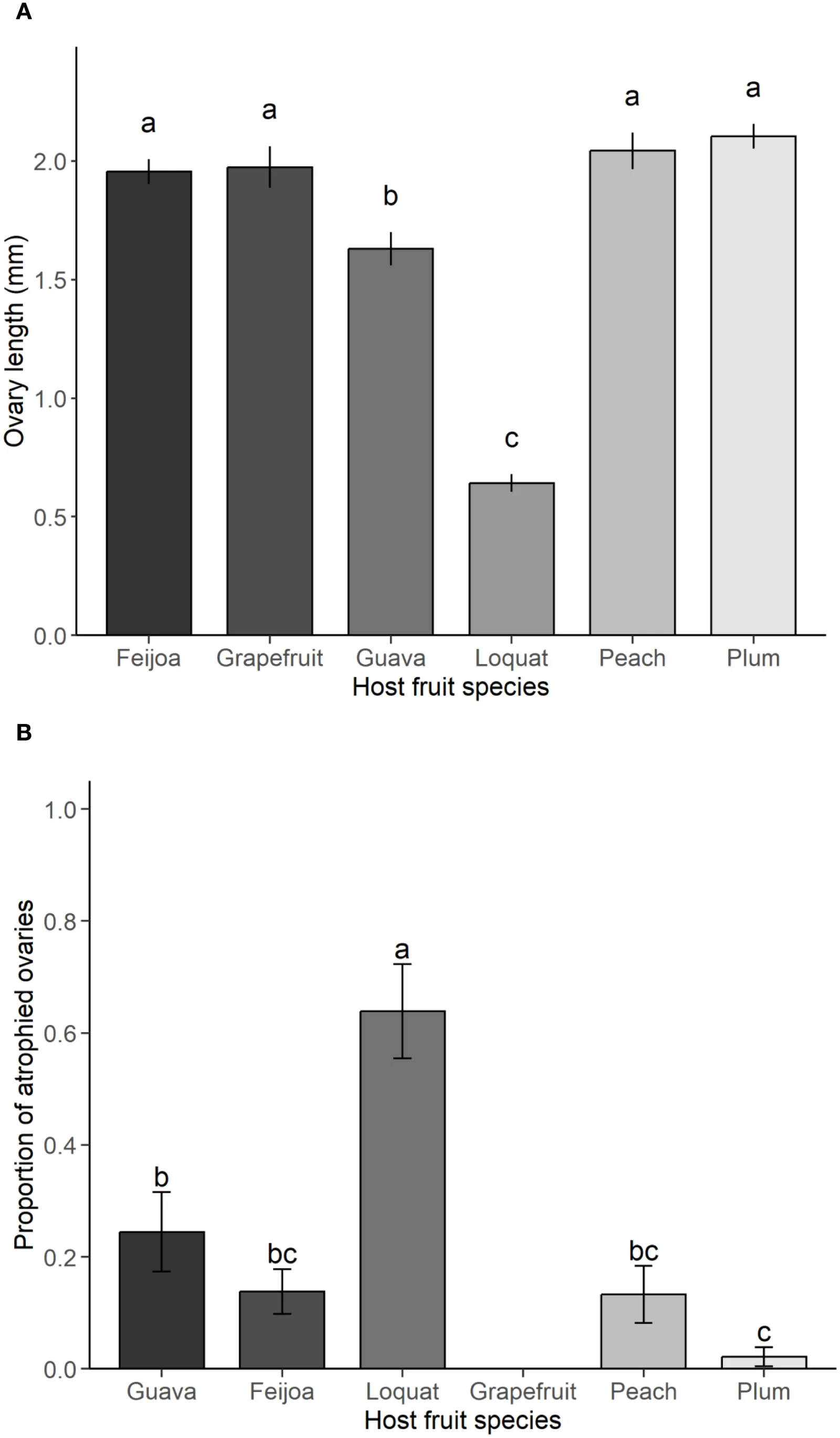
Effect of host species on ovary development in female *Anastrepha fraterculus*. The host species included were: *Psidium guajava* (guava), *Acca sellowiana* (feijoa), *Eriobotrya japonica* (loquat), *Citrus x paradisi* (grapefruit),, *Prunus persica* (peach), and *Prunus domestica* (plum). **(A)** Mean (± S.E.) estimated ovary length is shown in mm for each host species. **(B)** Proportion (± S.E.) of atrophied ovaries. Different letters indicate statistically significant differences among means (p < 0.05).

Ovary length in A. fraterculus differed among host species (likelihood ratio χ² = 193.12; d.f. = 5; p < 0.001). Females developing in peach, plum, feijoa, and grapefruit exhibited greater ovary length than those developing in guava, whereas ovary length was lowest in females developing in loquat among all host species (**Figure 6A**).

The proportion of atrophied ovaries in *A. fraterculus* females was also affected by host species (F = 13.63; d.f. = 5, 85; p < 0.001). Females developing as larvae in loquat and guava showed a significantly higher proportion of atrophied ovaries than those developing in plum. Females emerging from peach and feijoa exhibited intermediate proportions, whereas plum showed the lowest proportion of ovarian atrophy, and no atrophied ovaries were observed in females developing in grapefruit (**Figure 6B**).

### Sexual maturation

**Figure 7:**
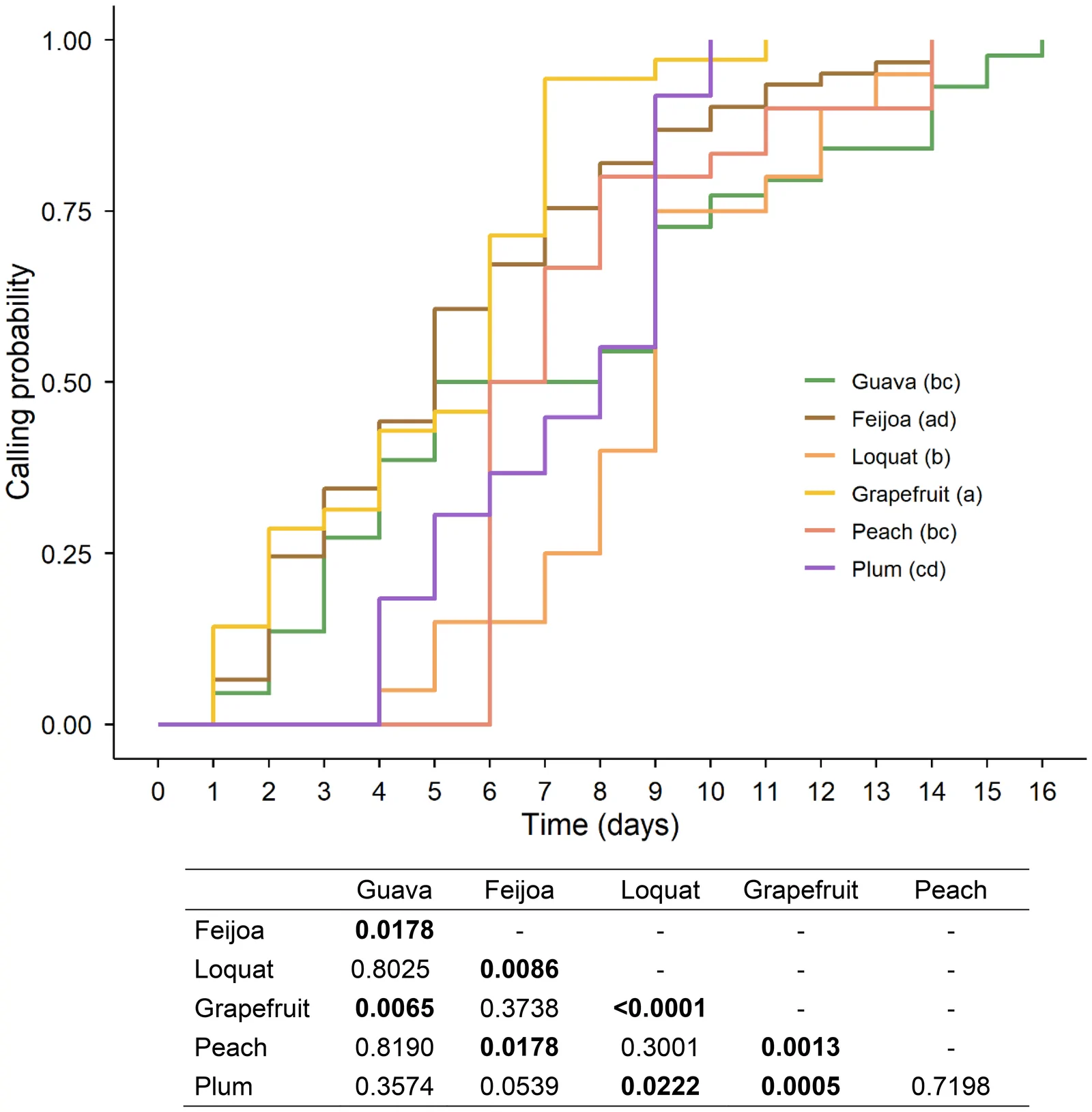
Effect of host fruit on the sexual maturation of *Anastrepha fraterculus* males. Males developed in *Psidium guajava* (guava), *Acca sellowiana* (feijoa), *Eriobotrya japonica* (loquat), *Citrus x paradisi* (grapefruit),, *Prunus persica* (peach), and *Prunus domestica* (plum). Kaplan–Meier curves show the cumulative proportion of males initiating sexual calling since emergence. Tables below the figure present multiple pairwise comparisons among host species. Differences with p < 0.05 were considered statistically significant (bold).

The onset of calling behavior was significantly affected by the host fruit species in which males developed (Likelihood-ratio, χ² = 27.18, d.f. = 5, p < 0.001). Males reared in grapefruit initiated sexual calling earliest, followed by those from feijoa, whereas males from plum and peach showed intermediate onset times. In contrast, males emerging from guava and loquat initiated sexual calling significantly later (**Figure 7**).

### Starvation resistance

**Figure 8:**
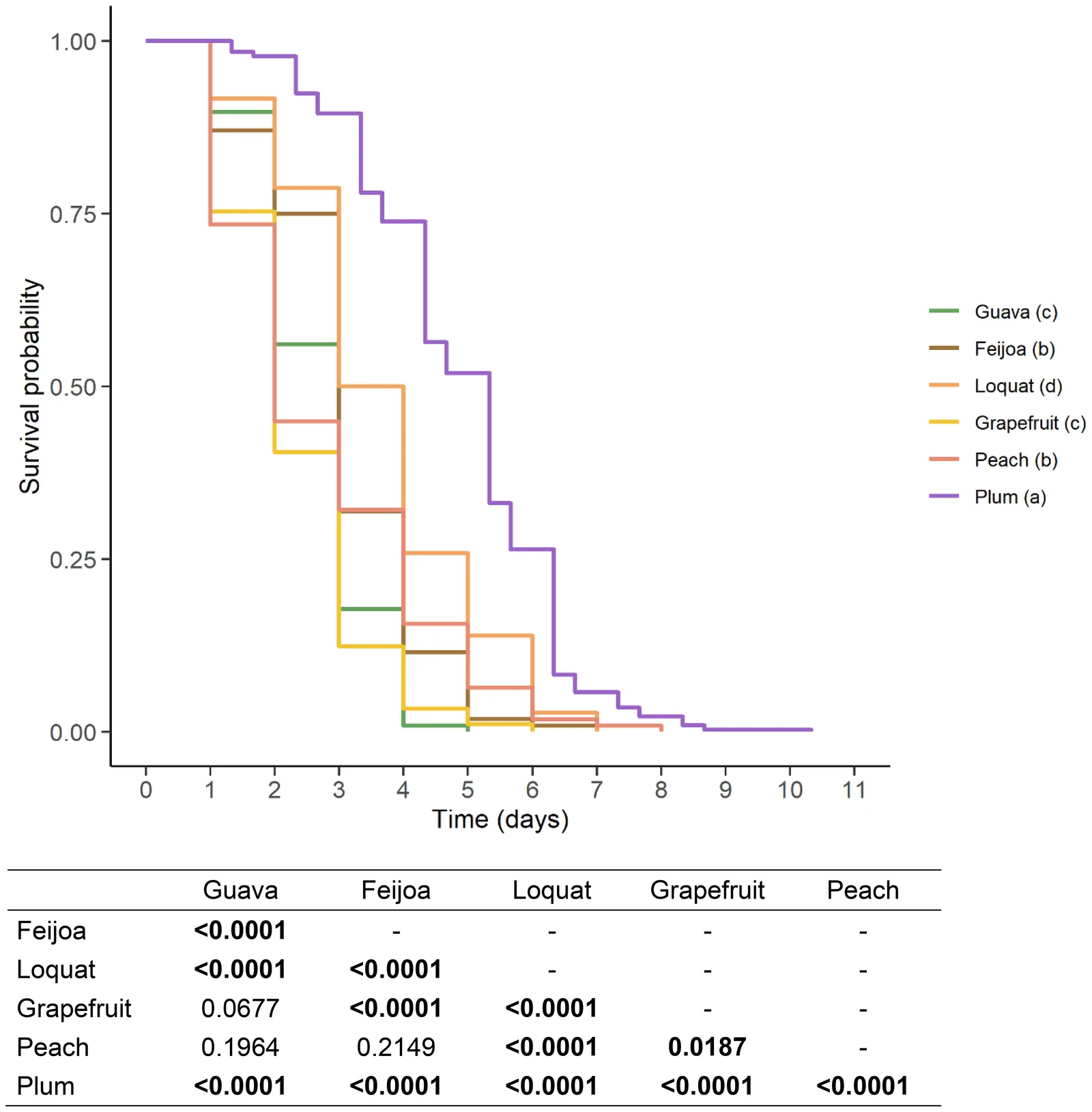
Starvation resistance of *Anastrepha fraterculus* adults that developed in different host species: *Psidium guajava* (guava), *Acca sellowiana* (feijoa), *Eriobotrya japonica* (loquat), *Citrus x paradisi* (grapefruit),, *Prunus persica* (peach), and *Prunus domestica* (plum). Kaplan–Meier curves show the proportion of individuals remaining alive under starvation conditions as a function of time since adult emergence. Tables below the figures present the results of multiple pairwise comparisons among host species. Differences with p < 0.05 were considered statistically significant (bold).

The mean longevity of starved *A. fraterculus* was significantly affected by the host species (likelihood-ratio χ² = 340.40; d.f. = 5; p < 0.001). There was no effect of sex (likelihood-ratio χ²= 0.38; d.f. = 1; p *=* 0.5339) or interaction between factors (likelihood-ratio χ² = 8.07; d.f. = 5; p = 0.153). Flies emerging from plum showed the highest starvation tolerance, followed by those from loquat, feijoa, and peach, while flies from guava and grapefruit exhibited the lowest tolerance (**Figure 8**).

### Heat map

**Figure 9:**
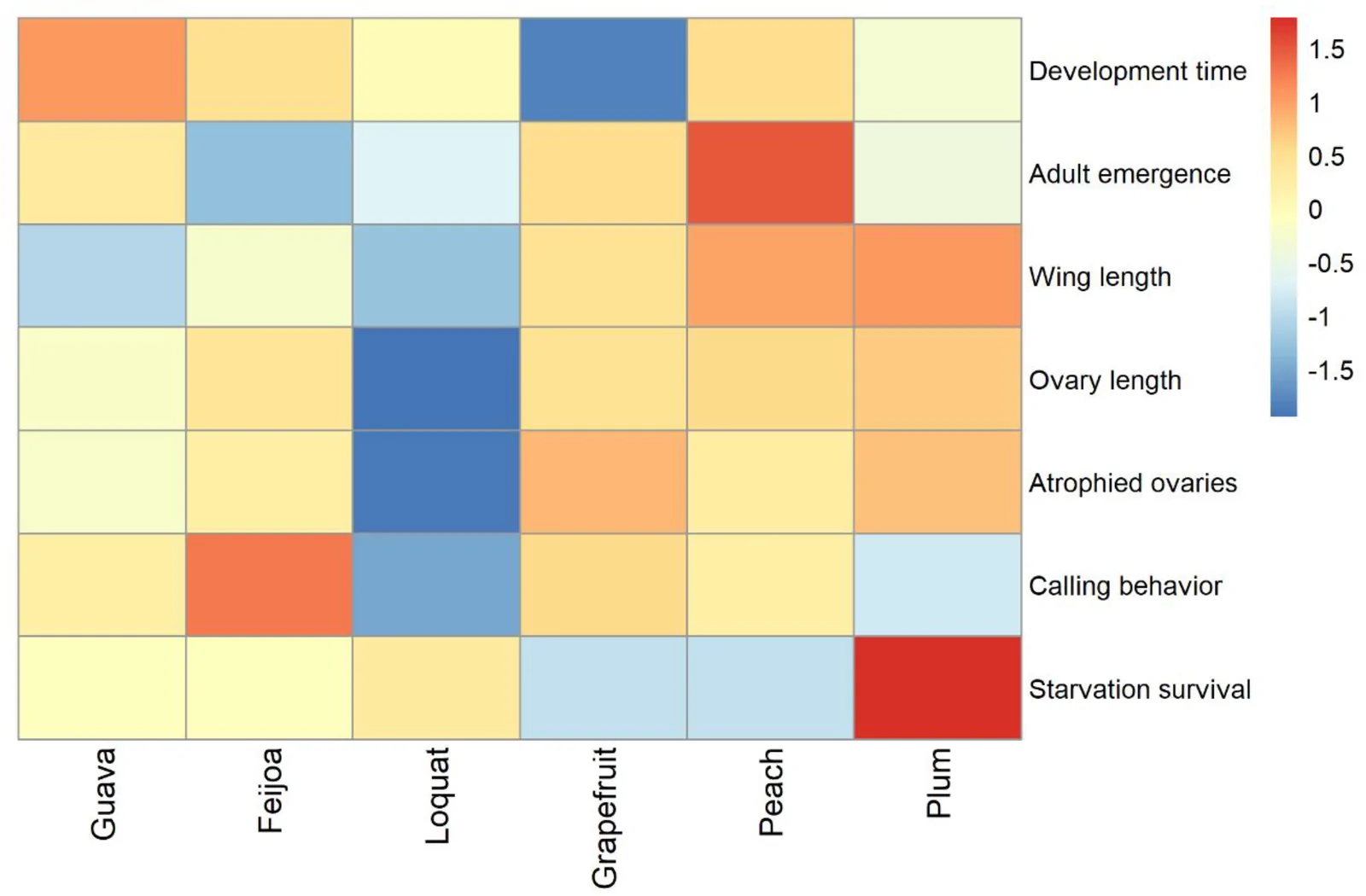
Heat map summarizing the relative performance of biological traits of *Anastrepha fraterculus* developed in different host species: *Psidium guajava* (guava), *Acca sellowiana* (feijoa), *Eriobotrya japonica* (loquat), *Citrus x paradisi* (grapefruit), *Prunus persica* (peach), and *Prunus domestica* (plum). Trait values were standardized as row-wise z-scores to allow comparisons across traits and host species. For traits in which lower values indicate better performance (development time, atrophied ovaries, and calling behavior), z-scores were multiplied by −1 prior to plotting. Thus, for all traits, higher values (red) represent better biological performance, whereas lower values (blue) indicate poorer performance. The heat map provides an overall comparison of offspring performance across host fruit species.

The heat map summarizing standardized biological traits revealed marked differences in performance among host fruits (**Figure 9**). Overall, plum and peach consistently exhibited higher relative performance across most traits, whereas loquat showed lower performance for several key biological variables.

#### Principal component analysis

**Figure 10:**
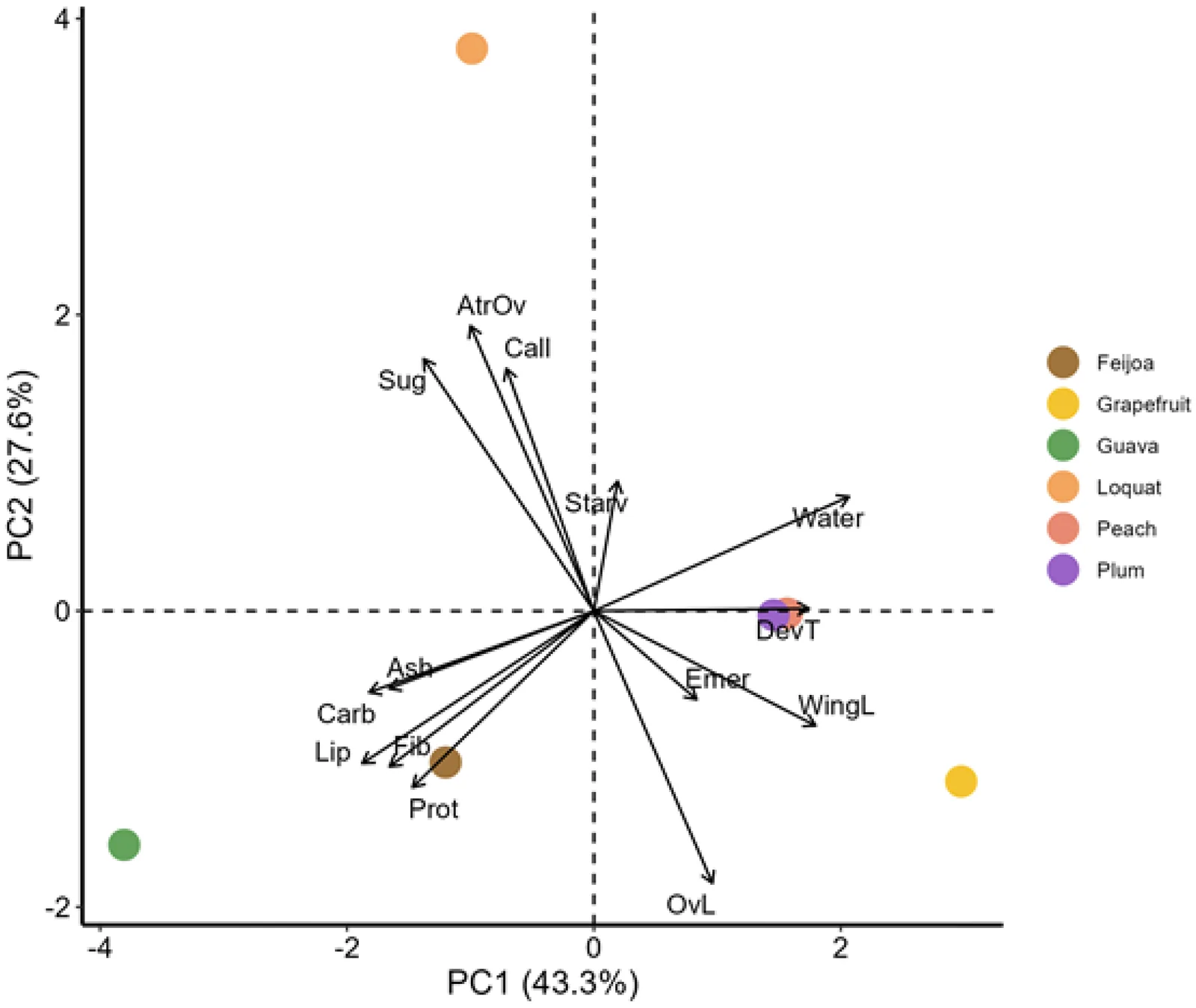
Principal component analysis (PCA) biplot showing the relationships between host fruit species nutritional composition and life-history traits of *Anastrepha fraterculus*. Host fruit species are displayed according to the mean values of nutritional variables and biological parameters measured in flies that developed and emerged from six host fruit species: *Psidium guajava* (guava), *Acca sellowiana* (feijoa), *Eriobotrya japonica* (loquat), *Citrus x paradisi (grapefruit)*, *Prunus persica* (peach), and *Prunus domestica* (plum). The percentages of variance explained by each principal component are indicated in parentheses. Variable abbreviations are: Water, water content; Prot, protein content; Lip, lipid content; Ash, ash content; Carb, carbohydrate content; Sug, sugar content; Fib, fiber content; Starv, starvation resistance; OvL, ovary length; DevT, developmental time; WingL, wing length; AtrOv, proportion of females with atrophied ovaries; Emer, adult emergence; and Call, male calling time.

In the PCA, the first two principal components explained 70.9% of the total variance, with PC1 accounting for 43.3% and PC2 accounting for 27.6% (**Figure 10**). PC1 was positively associated with water content, developmental time, and wing length, and negatively associated with protein, lipid, fiber, carbohydrate, and ash content. PC2 was positively associated with sugar content, the proportion of females with atrophied ovaries, and male calling behavior, and negatively associated with ovary length (**Figure 10**; **Supplementary Table 1**; **Supplementary Figure 1**). Host species were clearly differentiated in the PCA space. Guava was associated with higher protein, lipid, fiber, carbohydrate, and ash contents, whereas grapefruit was associated with higher water content, longer developmental time, and greater wing length. Loquat was associated with higher sugar content, increased male calling behavior, and a higher proportion of females with atrophied ovaries. In contrast, peach and plum largely overlapped, indicating similar nutritional and life-history profiles.

## Discussion

This study aimed to investigate how different fruit species affect larval development and adult life-history traits in *A. fraterculus*, a major fruit fly pest in South America. The results showed substantial variation in developmental time, pupal viability, adult size, reproductive indicators, and stress tolerance across fruit species, unveiling that no single host optimizes all aspects of performance. Although our experiment was not specifically designed to test the idea that native hosts necessarily provide better developmental conditions than exotic hosts (15, 33, 60), our results somewhat challenge this hypothesis and reveal a possible mismatch between infestation levels observed in the field in previous works (20, 61) and the actual suitability of those hosts in terms of offspring performance. Nonetheless, it is important to note that the performance parameters evaluated in this study were measured under controlled laboratory conditions, which do not incorporate several ecological factors that may strongly influence host suitability in nature, such as temperature fluctuations, desiccation risk, natural enemies, or seasonal constraints. Therefore, the adaptive value of native hosts in the field may not be fully captured by the laboratory-based performance metrics evaluated here. Another limitation of the present study is the lack of direct measurements of the actual degree of fruit maturity within each fruit species (e.g., sugar-to-acid ratio, pH, secondary metabolite profiles). Consequently, some level of variation may have existed among fruits categorized as “ripe,” potentially introducing additional variability into the observed results. The limitations mentioned above should be carefully considered when interpreting the study findings and the associated discussion.

In addition to nutritional quality, tephritid larval development is strongly influenced by competition (62–64). When larval density exceeds a threshold, it impacts various life-history traits, even at the adult stage (64–66). In our study, we kept larval densities low to avoid food limitation, aiming to isolate the effect of host fruit species. We verified this by analyzing the relationship between larval density and all measured parameters across fruit types. With few exceptions, larval density remained within non-competitive levels, so we are confident that differences among host species are not driven by density-related effects (data not shown).Fruit species significantly affected *A. fraterculus* larval developmental time. Guava allowed the fastest development, emerging as the most suitable host in this regard. Feijoa—another native Myrtaceae—and peach formed a second group, followed by loquat and plum. Grapefruit, one of the few citrus species consistently infested by *A. fraterculus* in Argentina (20, 61), resulted in the longest developmental time. The composition of the substrate where the larva develops—particularly lipid content—appears to play a central role in developmental duration (67, 68). In tephritid fruit flies, lipid-enriched substrates supported faster larval development. In agreement with this pattern, guava presented the highest lipid content among the tested substrates (**Table 1**). Similar patterns have been reported in other polyphagous fruit flies (67), although further investigation is needed to disentangle the specific contribution of lipids from other nutritional components. The PCA revealed a similar pattern, with guava associated with higher protein, lipid, fiber, carbohydrate, and ash contents, whereas grapefruit was associated with longer developmental time and greater wing length. This result supports the idea that no single host species provides optimal conditions for all performance-related traits.

Prolonged larval development may increase vulnerability to abiotic stress (temperature, rainfall) and biotic threats (natural enemies, parasites, pathogens), suggesting that grapefruit may be a risky host under field conditions. In *A. ludens*, Leyva et al. (69) reported longer larval developmental times in grapefruit than in peach. This difference was attributed to fruit characteristics: citrus pulp does not autolyze rapidly and remains moist for several weeks (no references were made to the effect of lipid content). This, contrary to positioning the grapefruit as a poor host, may imply that this species provides a protective environment that allows mature larvae to delay pupation. In contrast, peach pulp decomposes quickly, forcing larvae to exit and pupate earlier. This suggests that slower development may have advantages under certain conditions, for instance, when temperature decreases in wintertime in temperate areas, and flies could extend their larval stage overwintering inside the fruit, as reported for *C. capitata* (70). This would suggest that what may be considered an optimal host in terms of developmental time could depend on the temperature regimes and the time of the year each host becomes available.

Fruit species also affected pupal viability. Previous studies in tephritid fruit flies showed contrasting results regarding this parameter (33, 71–74). Here, pupal viability differed among fruit species, with peach, grapefruit, and guava yielding the highest adult emergence, followed by plum and loquat. These findings suggest that guava provides favorable conditions for the development of *A. fraterculus*, supporting consistent performance across immature stages. In contrast, a previous study in *A. fraterculus* by Bisognin et al. (33) found no effect of fruit species on pupal viability, though the host range differed from ours. Although both feijoa and guava (native Myrtaceae) promote rapid larval development, feijoa shows low pupal survival. Possible explanations may involve differences in defensive or toxic secondary compounds (e.g., terpenes or phenolic compounds) or sublethal nutritional limitations (e.g., deficiencies in essential nutrients or micronutrients, important for histogenesis and tissue development) that impair successful metamorphosis (75, 76). Notably, despite this apparent poor performance under laboratory conditions, feijoa is frequently found naturally infested by *A. fraterculus*. This suggests that factors other than offspring suitability may drive host use. For instance, feijoa may represent an abundant or phenologically reliable resource in the field, or females may exhibit limited ability to discriminate among hosts based on their suitability for larval development. These alternative hypotheses merit further investigation.

In females, larval host affected ovary size—a proxy for fecundity in fruit flies, as suggested by Hou et al. (52). Exotic hosts like peach, plum, and grapefruit—as well as feijoa—produced females with larger ovaries and lower rates of ovarian atrophy. This result aligns with that of wing length and also with earlier findings showing that larger body size in insects, including tephritid fruit flies, often correlates with greater fecundity (46, 77, 78). Protein content also has a profound effect on female reproductive output in tephritid fruit flies (46, 79, 80). Loquat produced females with smaller ovaries, a pattern that could be explained by its limited protein content (see **Table 1**). Nonetheless, guava, which has been reported to have high protein content, did not produce females with significantly larger ovaries than the rest of the hosts (except for loquat). So, the effect of the host species on ovary size, and presumably, on female fecundity, might be attributable to other factors. Consistent with this idea, the PCA showed that ovary length was not associated with the nutritional profile that characterized guava. This suggests that factors other than the measured nutritional components may contribute to female reproductive development.

Guava negatively affected ovary development, despite supporting fast development and high pupal viability. This rather unexpected result likely relates to the smaller body size of females from guava, which may indicate a cost of fast development. One hypothesis is that ancestral hosts like guava may have evolved chemical or structural defenses that limit larval performance as a response to herbivory. Specifically, guava pulp contains secondary metabolites such as phenols and tannins that function as potent anti-nutritional defenses (81). The defensive impact of these compounds is likely determined by their concentration relative to the energetic reward available; for instance, the sucrose-to-total phenolics ratio in guava is significantly lower (~2) than in exotic hosts like *P. persica* (~34), representing a much higher relative chemical burden for developing larvae (82). Consequently, metabolic energy may be diverted toward detoxification mechanisms rather than structural growth and reproductive maturation, which could explain the smaller adult size and impaired fecundity observed (81, 82). Such defenses may be absent from exotic hosts, which have a recent evolutionary history with *A. fraterculus*. Alternatively, guava presents higher ash content than the other fruits evaluated here (see **Table 1**), which may have contributed to reduced female size. High ash content is associated with a greater proportion of minerals and inorganic compounds that can alter osmotic balance and reduce nutrient absorption efficiency, ultimately impairing larval growth and resulting in lighter pupae and smaller adults (83, 84). The lack of one or more nutrients relevant to reproduction should not be discarded as a hypothesis. The PCA further supports this interpretation. Although guava was associated with higher protein and lipid contents, it was not associated with reproductive traits, suggesting that nutritional composition alone may not fully explain host quality. Other factors, such as secondary metabolites, mineral composition, or the physiological costs of detoxification, may also contribute to the patterns observed here. These ideas warrant further research.

In tephritid fruit flies, male reproductive traits like sexual maturation and pheromone signaling depend on protein intake during the larval and adult stages (85–88). Males reared on grapefruit and plum reached sexual maturity faster than those from other hosts. Interestingly, guava also supported rapid sexual maturation, despite producing smaller males and females, and negatively affecting ovary development. Previous studies showed that guava volatiles enhance sexual signaling in *A. fraterculus* males (54, 56, 89–91), suggesting that effects found here may not be purely nutritional but could involve sensory or physiological cues. In *C. capitata*, the host species also affected male sexual maturation, showing that the larval substrate may determine the reproductive output of tephritid fruit flies (92). Furthermore, the differences between the effect of host species in males and females suggest that host fruits can influence male and female traits in distinct ways, and that sex-specific responses to larval environment may shape adult behavior and fitness.

The ability of insects to withstand starvation or stress due to nutrient deprivation is closely linked to the quantity and quality of energy reserves accumulated as larvae (93–95). Adult starvation resistance also varied across hosts. Plum supported the highest survival under nutrient stress, followed by loquat and feijoa. Peach, guava, and grapefruit supported lower survival durations. Thus, hosts previously considered high-quality in terms of reproductive or developmental traits may be less effective in supporting stress resilience. Interestingly, larger wings—as seen in grapefruit and peach—did not translate into better starvation tolerance, suggesting that body size alone is not a reliable proxy for metabolic reserves.

In holometabolous insects, suitable larval diets promote protein and lipid accumulation, boosting adult emergence and fecundity (16, 17, 96, 97). As previously mentioned, protein is also essential for male reproductive development; males reared on protein-poor larval diets tend to show reduced sexual performance and delayed maturation (87, 98). In *Drosophila*, diets that are low in proteins and high in carbohydrates extend lifespan (18), but neither macronutrient alone ensures optimal longevity. Lipid reserves, in particular, may be crucial for adult performance (99). However, in our case, no single macronutrient component of the diet allowed to predict life-history outcomes. For instance, guava has a higher protein and lipid content than the other host species tested here, and should, theoretically, be the best host. Yet, apart from fast larval development, other life history parameters were lower in guava, which suggests a mismatch between protein content and fitness. As mentioned before, other traits associated with nutrient intake may have also played a role in nutrient acquisition during the larval stage.

## Conclusions

In sum, our results revealed a complex interaction between host use and performance-related traits in *A. fraterculus*. No fruit species consistently maximized all life-history traits, and the heatmap clearly highlights that trade-offs emerged between developmental time, size, fecundity, and stress resistance. The PCA further suggested that differences in the nutritional composition of host fruits did not fully account for the observed variation in life-history traits. Notably, guava and feijoa—two of the most infested fruits in nature—did not always yield superior performance in laboratory conditions. This mismatch suggests a decoupling between host preference and actual developmental quality, a phenomenon also documented in other phytophagous insects (5), which would argue against the preference-performance hypothesis. This could result from the complexity of the system, where exotic and native hosts become available at different times of the year. A possible, yet not tested, explanation is that different host species maximize distinct life-history traits at different times, such that no single host is universally optimal but, rather, hosts are selectively advantageous depending on seasonal conditions and the shifting demographic needs of the population (e.g., early-season hosts favoring reproductive output, whereas late-season hosts enhancing survival and stress tolerance). Nonetheless, it is important to keep in mind that in our work, preference was not directly assessed, and infestation in the field is not always a proxy for female preference. Also, the results obtained may not necessarily represent differences between host in terms of nutritional quality, as other factors, like oviposition opportunity, egg load, larval crowding, or internal fruit structure were not controlled in our experimental design, in which we only controlled the ratio of females to fruit surface. Understanding the ecological and evolutionary mechanisms behind these patterns is essential to interpret host use strategies in *A. fraterculus*, uncovering how populations establish and expand, which in turn is critical for developing effective management strategies. Future work combining behavioral assays and biochemical analyses of fruit composition will be critical to uncover the drivers of host selection and their implications for pest management.

## Data Availability

The raw data supporting the conclusions of this article will be made available by the authors, without undue reservation.

## Supplementary material

Supplementary Figure 1 Correlation circle from the principal component analysis (PCA) showing the correlations between nutritional variables and life-history traits of *Anastrepha fraterculus*. The percentages of variance explained by each principal component are indicated in parentheses. Variable abbreviations are: Water, water content; Prot, protein content; Lip, lipid content; Ash, ash content; Carb, carbohydrate content; Sug, sugar content; Fib, fiber content; Starv, starvation resistance; OvL, ovary length; DevT, developmental time; WingL, wing length; AtrOv, atrophied ovaries; Emer, adult emergence; and Call, male calling time.

Supplementary Table 1 Loadings of nutritional variables and life-history traits on the first two principal components (PC1 and PC2) of the principal component analysis (PCA). Loadings with absolute values ≥ 0.30 are shown in bold.
