# Supplementary material for: No single host is optimal: effects of fruit species on development and adult performance in Anastrepha fraterculus (Diptera: Tephritidae)
Source: Front Insect Sci. 2026 Aug 17;6:1832786. doi: 10.3389/finsc.2026.1832786 (PMC13526955; doi:10.3389/finsc.2026.1832786)
Supplement: Supplementary file 1 [file SupplementaryFile1.docx]

**Supplementary Figure 1**

**Supplementary Figure 1.** Correlation circle from the principal component analysis (PCA) showing the correlations between nutritional variables and life-history traits of *Anastrepha fraterculus*. The percentages of variance explained by each principal component are indicated in parentheses. Variable abbreviations are: Water, water content; Prot, protein content; Lip, lipid content; Ash, ash content; Carb, carbohydrate content; Sug, sugar content; Fib, fiber content; Starv, starvation resistance; OvL, ovary length; DevT, developmental time; WingL, wing length; AtrOv, atrophied ovaries; Emer, adult emergence; and Call, male calling time.
