# Supplementary material for: No single host is optimal: effects of fruit species on development and adult performance in Anastrepha fraterculus (Diptera: Tephritidae)
Source: Front Insect Sci. 2026 Aug 17;6:1832786. doi: 10.3389/finsc.2026.1832786 (PMC13526955; doi:10.3389/finsc.2026.1832786)
Supplement: Supplementary file 2 [file Table1.docx]

**Supplementary Table 1.** Loadings of nutritional variables and life-history traits on the first two principal components (PC1 and PC2) of the principal component analysis (PCA). Loadings with absolute values ≥ 0.30 are shown in bold.

| **Variable** | **PC1** | **PC2** |
| --- | --- | --- |
| Water | **0.377** | 0.177 |
| Protein | -0.268 | -0.272 |
| Lipid | **-0.342** | -0.235 |
| Ash | **-0.303** | -0.120 |
| Carbohydrate | **-0.332** | -0.126 |
| Sugar | -0.251 | **0.389** |
| Fiber | **-0.302** | -0.240 |
| Developmental time | **0.318** | 0.004 |
| Adult emergence | 0.152 | -0.137 |
| Wing length | **0.327** | -0.177 |
| Ovary length | 0.175 | -0.420 |
| Atrophied ovaries | -0.182 | **0.440** |
| Starvation resistance | 0.035 | 0.200 |
| Male calling time | -0.129 | **0.374** |
